# Insights Into Colorectal Carcinoma: A Comprehensive Review of MicroRNA Expression Patterns

**DOI:** 10.7759/cureus.56739

**Published:** 2024-03-22

**Authors:** Shweta Pandey, Akriti Jain, Sunita Vagha

**Affiliations:** 1 Pathology, Jawaharlal Nehru Medical College, Datta Meghe Institute of Higher Education and Research, Wardha, IND; 2 Pathology, Delhi State Cancer Institute, Delhi, IND

**Keywords:** personalized treatment, therapeutic targets, biomarkers, dysregulation, micrornas, colorectal carcinoma

## Abstract

Colorectal carcinoma (CRC) remains a significant contributor to cancer-related morbidity and mortality worldwide. MicroRNAs (miRNAs) have emerged as crucial regulators of gene expression and play critical roles in various biological processes, including carcinogenesis. This comprehensive review aims to elucidate the role of miRNAs in CRC by analyzing their expression patterns and functional implications. An extensive literature review identified dysregulated miRNAs associated with different stages of CRC progression, from initiation to metastasis. These miRNAs modulate key signaling pathways in tumor growth, invasion, and metastasis. Furthermore, we discuss the potential of miRNAs as diagnostic biomarkers and therapeutic targets in CRC management. Future research directions include elucidating the functional significance of dysregulated miRNAs using advanced experimental models and computational approaches and exploring the therapeutic potential of miRNA-based interventions in personalized treatment strategies for CRC patients. Collaboration among researchers, clinicians, and industry partners will be essential to translate these findings into clinically impactful interventions that improve patient outcomes in CRC.

## Introduction and background

Colorectal carcinoma (CRC), commonly known as colorectal cancer, is one of the leading causes of cancer-related mortality worldwide. It originates in the colon or rectum, often from precancerous polyps [1]. CRC progresses through stages, ranging from localized tumors to metastatic disease, posing significant challenges for effective treatment and management [2]. MicroRNAs (miRNAs) are small, noncoding RNA molecules that regulate gene expression [3]. Dysregulation of miRNAs has been implicated in various biological processes, including carcinogenesis. In cancer, aberrant miRNA expression can influence critical pathways in tumor initiation, progression, and metastasis [4]. Therefore, understanding the role of miRNAs in CRC holds promise for identifying novel diagnostic and therapeutic strategies [5].

This comprehensive review aims to provide insights into the role of miRNAs in CRC by analyzing their expression patterns. By synthesizing current knowledge from molecular studies, we aim to elucidate the significance of dysregulated miRNAs in the pathogenesis of CRC. Furthermore, we intend to discuss the potential clinical implications of miRNA-based biomarkers and therapeutic targets in managing colorectal cancer.

## Review

CRC: pathogenesis and molecular mechanisms

Genetic and Environmental Factors

**Figure 1:**
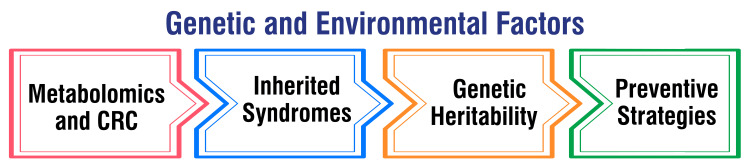
Genetic and environmental factors of colorectal carcinoma CRC: colorectal carcinoma The figure was self-created by the corresponding author Dr. Shweta Pandey.

Studies indicate that genetic heritability contributes to approximately 35% of colorectal cancer cases, underscoring a significant genetic predisposition to the disease [6]. Researchers have elucidated the interplay between genetic susceptibility, often manifested as single-nucleotide polymorphisms (SNPs), and environmental factors such as exercise, dietary patterns, and lifestyle choices in shaping CRC risk [6]. Furthermore, specific inherited syndromes like Lynch syndrome (hereditary nonpolyposis colorectal cancer) and familial adenomatous polyposis have been identified as factors significantly elevating the likelihood of developing colorectal cancer [7]. Environmental influences also play a critical role in CRC development, encompassing factors like aging, personal and familial history of CRC or adenomas, dietary habits, inflammatory bowel disease, sedentary behavior, obesity, and low-penetrance polymorphisms [7-9]. Metabolomics research has shed light on the impact of dietary metabolites, alcohol consumption, and genetic polymorphisms on CRC risk, indicating the importance of understanding these metabolic interactions for devising personalized therapeutic interventions [8]. In combating CRC, preventive strategies are paramount. Lifestyle modifications, including adopting a balanced diet, engaging in regular physical activity, and abstaining from smoking and excessive alcohol consumption, have been shown to mitigate up to 50% of CRC cases. Additionally, screening options tailored to individual environmental and genetic factors are indispensable for reducing both the incidence and mortality rates of colorectal cancer [9]. Genetic and environmental factors of CRC are shown in Figure 1.

Molecular Pathways Involved in CRC Development

Colorectal cancer is a complex ailment driven by diverse molecular pathways that play pivotal roles in its onset and advancement. Among the principal pathways implicated in CRC development are chromosomal instability (CIN), microsatellite instability (MSI), and the CPG island methylator phenotype (CIMP) [10]. CIN constitutes a fundamental pathway in CRC, characterized by genetic modifications leading to chromosomal abnormalities such as gains or losses of entire chromosomes or large segments. Mutations in essential genes like APC, KRAS, TP53, and BRAF are closely associated with tumorigenesis driven by CIN [10,11].

MSI stems from deficiencies in DNA mismatch repair mechanisms, resulting in mutations in microsatellite regions. This instability fosters CRC development by inactivating tumor suppressor genes, thus making it a pivotal pathway in CRC progression [11]. The CIMP involves the hypermethylation of CpG islands in gene promoters, culminating in gene silencing. This epigenetic alteration significantly contributes to CRC pathogenesis by silencing tumor suppressor genes [10].

Moreover, mutations in pivotal genes like APC activate the Wnt pathway, a critical occurrence in colorectal tumorigenesis [12]. The multistep genetic model proposed by Fearon and Vogelstein underscores the sequential events leading to CRC initiation, with APC inactivation representing a crucial early event [10]. Furthermore, mutations in signaling pathway components such as EGFR/MAPK, Notch, PI3K/AKT, and TGF-β have been associated with CRC development and progression [12]. Comprehending these molecular pathways is imperative for devising targeted therapies and personalized treatment strategies for CRC. Endeavors targeting specific constituents of these pathways with inhibitors are promising for pioneering therapeutic approaches to enhance patient outcomes in colorectal cancer treatment [12,13]. By unravelling the intricate molecular mechanisms underpinning CRC development, researchers strive to advance diagnostic techniques and therapeutic interventions tailored to individual molecular profiles, thus fostering more effective management of this disease.

Role of MiRNAs in CRC Pathogenesis

miRNAs play a pivotal role in the pathogenesis of CRC by orchestrating various cellular processes crucial for cancer development. The dysregulated expression of miRNAs detected in CRC significantly influences cell survival, apoptosis, epithelial-mesenchymal transition (EMT), and metastasis [14]. As inflammatory mediators, tumor oncogenes, or suppressors, these small noncoding RNAs modulate vital pathways such as Wnt/β-catenin and TGF-β signaling, profoundly impacting CRC tumorigenesis [14,15]. Inflammation and EMT are intricately linked processes in colorectal carcinogenesis, and miRNAs play a pivotal role in their regulation. By targeting inflammatory signaling molecules, miRNAs influence EMT, a crucial step in CRC progression. Notably, miRNAs regulate EMT by modulating the expression of critical molecules like E-cadherin, thereby shaping the metastatic potential of CRC [14].

The aberrant activation of the Wnt/β-catenin signaling pathway significantly drives colorectal tumorigenesis. Specific miRNAs, such as miR-135, directly target genes within this pathway, including APC, resulting in heightened Wnt signaling associated with advanced tumor grade and poor prognosis in CRC patients [15]. The therapeutic implications of miRNAs in CRC are profound, as they have emerged as potential targets for intervention. Manipulating miRNA expression levels can significantly impact cancer cell proliferation, invasion, and metastasis. For instance, restoring the expression miR-143 inhibits tumor cell growth and influences malignant transformation in colon cancer cells, underscoring the therapeutic potential of miRNA-based interventions in CRC management [14]. Understanding the intricate roles of miRNAs in CRC pathogenesis offers valuable insights into potential biomarkers and therapeutic strategies for this prevalent malignancy. Further research elucidating the specific functions of miRNAs in human carcinogenesis holds promise for identifying novel cancer diagnosis and treatment targets, ultimately leading to improved outcomes for patients with CRC [14].

Methods for miRNA expression profiling

High-Throughput Technologies

Next-generation sequencing (NGS) is a powerful tool enabling the comprehensive identification and quantification of miRNAs across the genome. Renowned for its high sensitivity, specificity, and versatility in analyzing diverse biospecimens, NGS offers unparalleled capabilities in miRNA research [16]. High-throughput sequencing (HTS), leveraging NGS methodologies, generates digital gene expression profiles suitable for miRNA expression profiling. Its genome-wide approach surmounts the limitations of array-based analyses, providing researchers with a more comprehensive understanding of miRNA expression patterns [17]. Microarray platforms, though offering a cost-effective and efficient means of miRNA expression profiling, may exhibit comparatively lower sensitivity and dynamic range than NGS. Nevertheless, they remain valuable in the miRNA research toolkit [18]. Reverse transcription quantitative polymerase chain reaction (qRT-PCR) emerges as a highly sensitive and specific method for miRNA expression profiling. Leveraging known concentrations of synthetic miRNA oligos, qRT-PCR facilitates absolute miRNA quantification, thus enhancing the precision of miRNA analysis [18]. Bioinformatics tools are crucial in analyzing the vast datasets generated by high-throughput technologies. These tools aid in tasks such as mapping reads to miRNA databases, quantifying isoforms, and identifying differentially expressed miRNAs through seed analysis, enabling comprehensive exploration of miRNA expression profiles [17]. High-throughput technologies have revolutionized our understanding of miRNA expression patterns and their involvement in various biological processes. They are indispensable resources for researchers investigating miRNA expression profiling and exploring its potential applications in diagnostics, prognostics, and therapeutics.

Data Analysis Approaches

Quality assessment of raw sequencing data files is a critical initial step in miRNA expression profiling, ensuring the reliability of subsequent analyses. Following this, reference-based alignment is conducted to map the reads accurately to the reference genome or miRNA database, facilitating precise quantification of miRNA expression levels [19]. Once alignment is completed, counts are generated to quantify miRNA expression levels, providing valuable insights into miRNA abundance. This process involves counting the transcripts corresponding to each miRNA, offering essential information for downstream analyses [19]. Researchers often analyze differential expression to identify miRNAs exhibiting significant expression differences between experimental conditions. Fold-change values are commonly utilized to categorize miRNAs as upregulated (fold-change ≥ 1.25) or downregulated (fold-change ≤ 0.8) [17]. Validation of HTS results is crucial through comparison with other platforms such as microarrays and quantitative PCR (qPCR). This validation ensures the consistency and reliability of findings across different profiling methods [17]. Bioinformatics tools play a vital role in miRNA expression profiling data analysis, facilitating the processing of raw sequencing data, differential expression analysis, and effective interpretation of results [19]. Researchers must consider various platform-specific factors and study goals when selecting a data analysis approach. Factors such as dynamic range requirements, method specificity, budget constraints for bioinformatic analysis, time investment, and the necessity for extensive normalization procedures should be considered [20]. Researchers can effectively analyze miRNA expression data obtained through various profiling methods by employing these data analysis approaches and considering platform-specific factors. This comprehensive analysis enables the exploration of miRNA regulation and its implications in biological processes and diseases like cancer.

MiRNA expression patterns in CRC

Dysregulated MiRNAs Associated with Tumor Initiation

Dysregulation of miRNA expression in CRC is intricately linked to processes driving tumor growth, metastasis, and heightened malignancy of tumor cells [21]. Studies have pinpointed specific dysregulated miRNAs in CRC, including miR-490-3p, miR-628-3p/-5p, miR-1297, miR-3151, miR-3163, and miR-3622a-5p (downregulated) and miR-105, miR-549, miR-1269, miR-1827, miR-3144-3p, miR-3177, miR-3180-3p, and miR-4326 (upregulated) [22]. These dysregulated miRNAs are promising biomarkers for CRC diagnosis and prognosis, offering a robust training set for validation in larger cohort studies [22]. From a clinical standpoint, a spectrum of dysregulated miRNAs has been associated with CRC genesis, progression, and response to therapy. Rigorous evaluation across multiple independent cohorts is imperative for realistic assessments of the utility of miRNAs in CRC diagnosis and prognosis [23]. In CRC, miRNAs function as oncogenes or tumor suppressors, influencing critical cellular processes such as proliferation, apoptosis, angiogenesis, and metastasis. The dysregulation of these molecules disrupts the normal expression levels of target mRNAs, impacting protein production [24]. The diagnostic potential of specific miRNA profiles presents a promising avenue for noninvasive early detection and risk assessment in CRC. Additionally, miRNA-based therapies offer a compelling strategy for targeted cancer treatment through modulation of miRNA expression [24].

MiRNAs Involved in Tumor Progression and Metastasis

Metastamir, a subset of miRNAs, has emerged as a pivotal regulator of metastasis, exhibiting the capability to either promote or inhibit metastatic processes through diverse mechanisms. These mechanisms encompass the regulation of migration, invasion, colonization, cancer stem cell properties, EMT, and modulation of the tumor microenvironment [25]. The intricate regulation of metastasis involves miRNAs orchestrating different stages, including local invasion, anoikic resistance, extravasation, and colonization. Through their regulatory functions, miRNAs exert significant influence over the metastatic cascade [26]. MiRNAs play a crucial role in sculpting the tumor microenvironment by mediating interactions between tumor and nontumoral cells. This interaction influences critical processes such as immunosuppression and angiogenesis, which are indispensable for metastasis [27]. EMT and its reverse process, mesenchymal-epithelial transition (MET), are finely tuned by miRNAs, regulating cancer cell motility, invasiveness, and colonization at distant sites. This dynamic control over EMT/MET processes underscores the importance of miRNAs in metastatic dissemination [26,27]. Specific miRNAs have emerged as attractive therapeutic targets for cancer treatment owing to their pivotal roles in metastasis regulation. Targeting these miRNAs holds promise for novel therapeutic interventions to mitigate metastatic spread [25,28]. Moreover, dysregulated miRNAs have been implicated in affecting critical aspects of cancer progression, including invasion and metastasis, thus holding potential as diagnostic and prognostic biomarkers for cancer. Their aberrant expression profiles offer valuable insights into disease prognosis and aid in developing personalized treatment strategies [29]. These collective findings underscore the multifaceted role of miRNAs in driving tumor progression and metastasis in CRC and other cancers. They illuminate potential avenues for targeted therapies and diagnostic strategies to combat metastatic disease.

MiRNAs as Prognostic and Predictive Biomarkers

MiRNAs have emerged as promising candidates for diagnostic and prognostic biomarkers in high-incidence cancers like CRC, owing to their association with abnormal expression profiles across various malignancies. These molecular signatures offer potential utility in cancer screening and prognostic assessment, providing valuable insights into disease progression and patient outcomes [30]. One of the critical advantages of miRNAs lies in their remarkable stability and detectability in clinical tissue specimens. Their robustness allows for easy extraction from diverse sources, including blood, facilitating a minimally invasive approach to cancer diagnosis and monitoring. This characteristic makes miRNAs attractive targets for biomarker discovery and clinical application [31,32]. In CRC patients, circulating miRNAs have garnered attention as potential prognostic and predictive biomarkers. Notably, miR-21 and other circulating miRNAs have shown promise in predicting clinical outcomes and treatment response, offering valuable prognostic insights and guiding therapeutic decision-making in CRC management [32]. Furthermore, miRNA expression patterns have been linked to predicting tumor mutational burden in CRC, which holds significance in selecting appropriate immunotherapy strategies. This association underscores the potential of miRNAs as predictive biomarkers, facilitating personalized treatment approaches tailored to individual tumor characteristics [32]. In addition to their prognostic value, miRNAs serve as predictive biomarkers for therapeutic response in various cancer types, including early breast cancer. Their stability, detectability, and association with treatment response make them valuable tools in guiding personalized medicine approaches, optimizing treatment outcomes, and minimizing adverse effects [31]. Moreover, colorectal cancer models have revealed genotype-specific patterns of miRNA expression, emphasizing the potential of specific miRNAs such as miR-155 as diagnostic and prognostic markers. This genetic specificity offers opportunities for refining risk stratification and treatment selection in CRC patients, paving the way for personalized therapeutic interventions [33].

Functional implications of dysregulated miRNAs

Target Genes and Pathways Regulated by Dysregulated MiRNAs

Dysregulated miRNAs have the potential to profoundly impact various cellular processes and pathways, exerting influence on gene expression and contributing to the development of diseases. In oral squamous cell carcinoma (OSCC), dysregulated miRNAs, such as let-7d, miR-1, and miR-125b-5p, among others, have been implicated in the disease's pathogenesis [34]. Similarly, in liver cancer, miR-124 has been identified as inhibiting self-renewal ability and associated with prognostic survival in hepatocellular carcinoma (HCC) [34]. Furthermore, dysregulated miRNAs play a role in neurometabolic diseases like propionic acidemia, impacting cellular physiology and disease development [35]. The manipulation of miRNA function holds considerable promise for clinical applications, including personalized medicine and developing novel therapeutic approaches [35]. Target genes regulated by dysregulated miRNAs involve diverse pathways, including cell proliferation, apoptosis, and metabolism. For instance, miRNA target genes regulate similar pathways in lung cancer and chronic obstructive pulmonary disease (COPD) [35]. Similarly, in neurodegenerative diseases like amyotrophic lateral sclerosis (ALS), dysregulation of miRNAs and target gene networks have been implicated in the disease's pathogenesis [36]. Integrative analysis of miRNA and mRNA expression data can unveil disrupted pathways regulated by miRNAs in cancer, identifying specific relationships between miRNAs and pathways disrupted in cancer [37].

Experimental Models to Study MiRNA Function in CRC

Various experimental models are employed to study miRNA function in CRC, aiming to elucidate the role of miRNAs in cancer development and progression. Animal models, such as mice, have been instrumental in identifying specific miRNAs like miR-33a as tumor suppressors and investigating their impact on tumor growth [24]. Concurrently, research has utilized human cell lines, including HT29, HCT116, HepG2, Huh7, and HEK293, to delve into miRNA-mediated regulation of gene expression in colon carcinoma. Studies have implicated the Let-7 family in downregulating specific genes in colon cancer cells, shedding light on the intricate molecular mechanisms underlying disease pathogenesis [24]. Furthermore, emphasis has been placed on establishing miRNA expression profiles in patient cohorts to uncover potential biomarkers and therapeutic targets for colorectal cancer [38]. By analyzing miRNA expression patterns in CRC tumors and normal tissues, researchers have pinpointed specific miRNAs like miR-24-3p as candidate regulators of gene expression across different mutational contexts [39]. These endeavors contribute to a deeper understanding of the functional implications of dysregulated miRNAs in CRC and provide a platform for exploring novel therapeutic approaches targeting miRNA expression.

Clinical applications and therapeutic potential

MiRNAs as Diagnostic Biomarkers

miRNAs have garnered attention as promising diagnostic biomarkers for colorectal cancer owing to their distinct expression patterns in cancerous and healthy cells [32]. Multiple studies have pinpointed specific miRNAs that hold potential as diagnostic and prognostic indicators in CRC patients [40]. These identified miRNAs exhibit the ability to discern individuals with CRC from healthy counterparts, offering prospects for early detection and risk assessment [41]. A systematic review and meta-analysis conducted by researchers affiliated with the National Center for Biotechnology Information (NCBI) underscored the potential of circulating miRNAs as diagnostic biomarkers across various diseases, including cancer [42]. Focusing on CRC, the study delved into the relevance of miRNAs as diagnostic, prognostic, and therapeutic targets [32]. Similarly, a review published in MDPI emphasized the pivotal roles of miRNAs in CRC and their viability as diagnostic biomarkers, stressing the necessity of comprehending their functions to devise more efficacious treatment modalities [43]. In alignment with these findings, a systematic review and meta-analysis published in Nature elucidated numerous miRNAs linked to colorectal carcinogenesis and their capacity to differentiate CRC patients from healthy individuals [41]. This comprehensive study further bolsters the potential of miRNAs as diagnostic biomarkers for CRC. Due to their discernible expression disparities between cancerous and normal cells, miRNAs present promise as diagnostic biomarkers for CRC. Further validation and refinement of these findings are imperative to develop more precise and reliable diagnostic tools based on miRNA expression profiles.

Therapeutic Targeting of Dysregulated MiRNAs

Therapeutic targeting of dysregulated miRNAs represents a promising frontier in cancer treatment, including colorectal cancer. MiRNAs are pivotal in regulating gene expression and cellular functions within cancer cells. Studies have elucidated that cancer cells often upregulate specific miRNAs to suppress gene expression in cell cycle regulation, thereby facilitating cancer progression [44]. Dysregulated miRNAs can influence various hallmarks of cancer, including sustaining proliferative signaling, evading growth suppressors, resisting cell death, promoting invasion and metastasis, and inducing angiogenesis [29]. In the realm of therapeutic targeting, multiple approaches have been explored. One strategy involves utilizing miRNA mimics to restore the function of tumor-suppressing miRNAs that are downregulated in cancer cells. These mimics, which are chemically modified double-stranded RNA molecules, mimic endogenous mature miRNAs and can impede cellular pathways that support oncogenesis [45]. Furthermore, developing small-molecule inhibitors and compounds provides another avenue for modulating the miRNA pathway, opening new possibilities for therapeutic intervention [45]. Additional strategies include miRNA masking, where sequences with perfect complementarity to miRNA binding sites on mRNA prevent miRNA binding and regulate gene expression [45]. Overall, therapeutic targeting of dysregulated miRNAs holds great promise for developing novel treatment strategies for colorectal cancer and other cancer types. However, further research and clinical trials are imperative to validate the efficacy and safety of these approaches in clinical settings.

Challenges and Future Directions in MiRNA-Based Therapeutics

Target identification: Identifying suitable miRNA targets is critical in developing effective therapy. While HTS and bioinformatics tools facilitate this process, further refinement is necessary to ensure specificity and efficacy [46]. Achieving precise target identification is pivotal for minimizing off-target effects and maximizing therapeutic benefits.

Efficiency of miRNA inhibitors: The moderate efficacy of miRNA inhibitors, such as antisense oligonucleotides (antagomirs, antimirs) and genetic knockouts, presents a significant challenge in miRNA therapeutics [47]. Enhancing the efficiency of these inhibitors is imperative for their clinical applicability. Strategies to improve inhibitor design, delivery methods, and pharmacokinetic properties are essential to enhance their therapeutic effectiveness.

Cell type-specific delivery: Efficient delivery of miRNA therapeutics to target tissues remains a significant obstacle. Various delivery systems, including nanoparticles, liposomes, and viral vectors, have been explored to address this challenge [48]. However, further optimization is necessary to ensure targeted delivery with minimal off-target effects and toxicity. Developing delivery systems that can navigate biological barriers and selectively target cancer cells holds promise for enhancing the efficacy and safety of miRNA-based therapies.

Adverse outcomes: The potential for adverse outcomes, such as off-target effects and immune responses, necessitates careful consideration while developing miRNA therapeutics [49]. Strategies to minimize off-target effects, enhance target specificity, and mitigate immune responses are crucial for ensuring the safety and efficacy of miRNA-based treatments. Preclinical studies and rigorous safety assessments are essential to identify and address potential adverse effects before advancing to clinical trials.

Regulatory approval: The regulatory approval process for miRNA-based therapies is intricate and time-consuming. Collaboration between academia, industry, and regulatory agencies is indispensable to streamline the regulatory pathway and expedite the development of safe and effective miRNA therapeutics [50]. Clear guidelines and robust regulatory frameworks are essential to ensure the rigorous evaluation of miRNA-based treatments while facilitating timely patient access.

Multidisciplinary approach: Overcoming the challenges in miRNA therapeutics requires a multidisciplinary approach involving biologists, chemists, pharmacologists, and clinicians [51]. Collaboration between diverse disciplines enables a comprehensive understanding of miRNA biology, optimal design of therapeutic agents, and translation of preclinical findings into clinical applications. By fostering interdisciplinary collaboration, researchers can harness the full potential of miRNA-based therapies to address unmet medical needs in cancer treatment.

## Conclusions

In conclusion, our comprehensive review of miRNA expression patterns in CRC has revealed several significant findings. We have identified a multitude of dysregulated miRNAs associated with different stages of colorectal cancer, from initiation to metastasis. These miRNAs exert regulatory effects on critical signaling pathways involved in tumor growth, invasion, and immune evasion. Additionally, our analysis underscores the potential of miRNAs as diagnostic biomarkers and therapeutic targets in colorectal cancer management. Future research endeavors should focus on elucidating the functional significance of dysregulated miRNAs using advanced experimental models and computational approaches. Furthermore, exploring the therapeutic potential of miRNA-based interventions in preclinical and clinical settings could lead to personalized treatment strategies for patients with colorectal cancer. Collaborative efforts among researchers, clinicians, and industry partners will be pivotal in translating these discoveries into clinically impactful interventions that ultimately improve patient outcomes in CRC.
